# Exosomes as Emerging Drug Delivery and Diagnostic Modality for Breast Cancer: Recent Advances in Isolation and Application

**DOI:** 10.3390/cancers14061435

**Published:** 2022-03-10

**Authors:** Dulla Naveen Kumar, Aiswarya Chaudhuri, Farrukh Aqil, Deepa Dehari, Radha Munagala, Sanjay Singh, Ramesh C. Gupta, Ashish Kumar Agrawal

**Affiliations:** 1 Department of Pharmaceutical Engineering & Technology, Indian Institute of Technology (BHU), Varanasi 221005, India; dullanaveenkr.rs.phe20@itbhu.ac.in (D.N.K.); aiswaryachaudhuri.rs.phe20@itbhu.ac.in (A.C.); deepa.dehari.rs.phe18@itbhu.ac.in (D.D.); ssingh.phe@iitbhu.ac.in (S.S.); 2 James Graham Brown Cancer Center, University of Louisville, Louisville, KY 40202, USA; farrukh.aqil@louisville.edu (F.A.); radha.munagala@louisville.edu (R.M.); rcgupta@louisville.edu (R.C.G.); 3 Department of Medicine, University of Louisville, Louisville, KY 40202, USA; 4 Department of Pharmacology and Toxicology, Baxter II Research Building, University of Louisville, Louisville, KY 40202, USA

**Keywords:** exosomes, breast cancer, diagnosis, drug delivery, chemotherapy, biomarker, targeted therapy

## Abstract

**Simple Summary:**

Breast cancer is considered the second most occurring cancer after lung cancer globally. The treatment for breast cancer includes surgery, radiation, chemotherapy, and immunotherapy. Among these therapies, chemotherapy has remained the backbone of the cancer treatment, however, chemotherapy is associated with various side effects like loss of hair, vomiting, nephrotoxicity, and neurotoxicity. Such drawbacks led to the emergence of a novel drug delivery system that will bypass the listed side effects and deliver the drugs to the target site. Exosomes are considered as an emerging novel drug delivery system derived from biological fluids such as blood, urine, saliva, etc., and range between 50 and 150 nm. In this review, we summarized various ways by which the exosomes are being isolated from these biological fluids, along with different drug loading techniques and their application as drug delivery carriers and diagnostic tools in the management of breast cancer.

**Abstract:**

Breast cancer (BC) is the most common type of malignancy which covers almost one-fourth of all the cancers diagnosed in women. Conventionally, chemo-, hormonal-, immune-, surgery, and radiotherapy are the clinically available therapies for BC. However, toxicity and other related adverse effects are still the major challenges. A variety of nano platforms have been reported to overcome these limitations, among them, exosomes provide a versatile platform not only for the diagnosis but also as a delivery vehicle for drugs. Exosomes are biological nanovesicles made up of a lipidic bilayer and known for cell-to-cell communication. Exosomes have been reported to be present in almost all bodily fluids, *viz.,* blood, milk, urine, saliva, pancreatic juice, bile, peritoneal, and cerebrospinal fluid. Such characteristics of exosomes have attracted immense interest in cancer diagnosis and therapy. They can deliver bioactive moieties such as protein, lipids, hydrophilic as well as hydrophobic drugs, various RNAs to both distant and nearby recipient cells as well as have specific biological markers. By considering the growing interest of the scientific community in this field, we comprehensively compiled the information about the biogenesis of exosomes, various isolation methods, the drug loading techniques, and their diverse applications in breast cancer diagnosis and therapy along with ongoing clinical trials which will assist future scientific endeavors in a more organized direction.

## 1. Introduction

In 2020, there have been an estimated 2,261,419 new cases (11.7% of all cancer types), and 684,996 mortalities (6.9% of all cancer types) caused by breast cancer (BC), globally. BC, among other cancers, remains the leading cause of cancer-related mortality among women [1]. BC is divided mainly into two types, invasive and non-invasive which is based on their spreading ability to the different organs. Invasive BC is more common and is further subdivided into invasive ductal carcinoma and invasive lobular carcinoma based on the origin of the tumor. BC is also divided into triple-negative breast cancer (TNBC), luminal (A and B), and human epidermal growth factor receptor (HER2) based on the status of the hormonal receptor. Among these, TNBC is highly aggressive with absence of estrogen receptor (ER), progesterone receptor (PR), and HER2 on the tumor [2]. Depending upon the stage of cancer, treatment modalities, either alone or in combination, include surgical removal of the tumor, radiation therapy, chemotherapy, and hormonal therapy. Among these, chemotherapy is considered the first-line treatment choice [3] even after having severe dose-dependent systemic toxicity and a lack of specificity [4]. Tremendous efforts have been made to overcome these limitations either by adopting the alternative route of administration or site-specific delivery strategies for the existing chemo drugs.

For the past decade, extensive research has been carried out in the area of nanotechnology and targeted therapy. Some of these candidates were approved for clinics based upon the risk over benefit ratio for clinically available formulations. However, high cost, poor specificity, and elimination by the reticuloendothelial system (RES) are some of the limitations that narrowed down the wide application of nanotechnology [5]. To further improve the efficacy, and overcome the present limitations, various novel nano-carriers have emerged; extracellular vesicles (EVs) are one of them. EVs are nano-sized, bilayer membrane-bound vesicles that are naturally secreted by the cells into the extracellular environment. The EVs are further divided into three different types based on their molecular genesis and composition; (i) apoptotic bodies, (ii) exosomes, and (iii) microvesicles, which are naturally loaded with a variety of cargos and rich in disease-specific biomarkers [6]].

**Figure 1:**
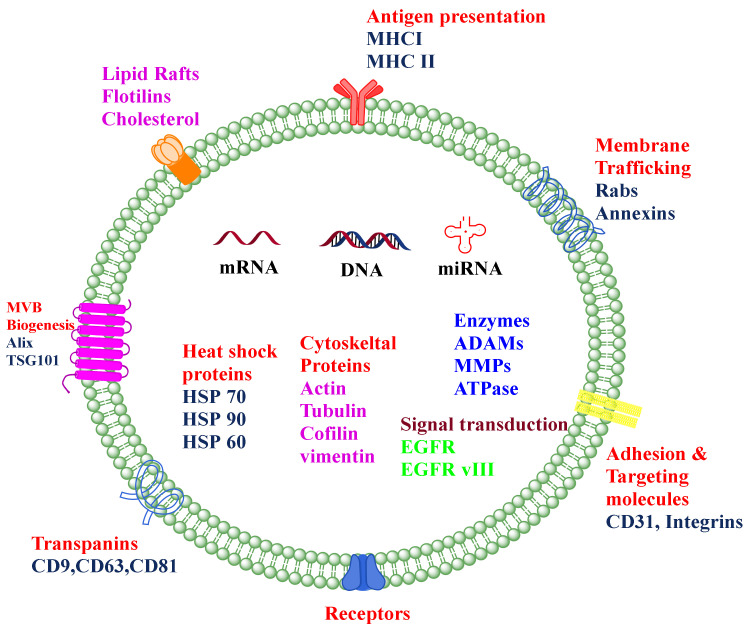
The composition of the typical exosome. Exosomes are nano-sized extracellular vesicles composed of a phospholipid bilayer, proteins such as TSG 101, Integrins, Alix, HSP, nucleic acids such as miRNA, mRNA, DNA, and many other receptors.

**Figure 2:**
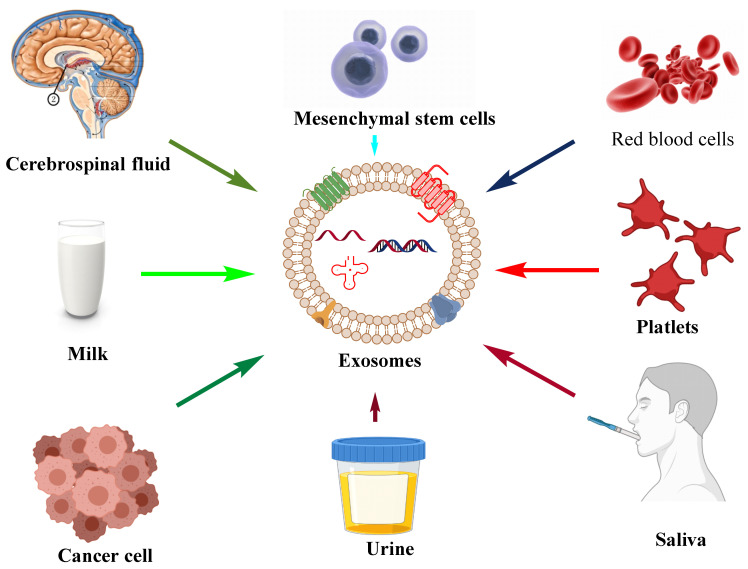
Different sources, *viz.,* urine, saliva, cancer cells, platelets, milk, cerebrospinal fluid, mesenchymal stem cells, and red blood cells majorly reported for the isolation of exosomes and their use in diagnosis and drug delivery.

The apoptotic bodies originate from the plasma membrane at the time of the cell death process, i.e., apoptosis. They are heterogeneous-shaped vesicles in the size range of 50–5000 nm. Apoptotic bodies contain DNA, RNA, histone, cell signaling molecules (messengers), nuclear fractions, and cell organelles. Apoptotic bodies display high expression of Annexin V (biomarker for apoptotic bodies) in their membrane [7]. On the other hand, the microvesicles, which are also known as exovesicles, ectosomes, shedding vehicles, microparticles, and plasma membrane-originated vesicles, either originate from the outward budding of the plasma membrane or by the fusion of the plasma membrane. The size of microvesicles ranges from 50 to 1000 nm which may contain a variety of cargos, *viz.,* miRNA, mRNA, other non-coding RNA, receptor proteins, membrane protein, and cytoplasmic protein [8]. Exosomes originate from multivesicular bodies (MVB) and are formed by the inward budding of late endosomes. The representative diagram of a typical exosome is depicted in Figure 1. Their size ranges between 30 and 150 nm and is secreted by various biological fluids such as blood, tears, saliva, cerebrospinal fluid, and milk. They contain mRNA, miRNA, other non-coding RNA, immune protein (major histocompatibility complex (MHC)), cytoplasmic, receptor, and membrane proteins [9,10]. The comparison of different types of EVs and different sources of exosomes are represented in Table 1 and Figure 2, respectively.

The growing interest in EVs, especially on exosomes, is not only represented by the increasing number of scientific works on exosomes but also by the increasing number of scientific association portals and databases such as

1. ISEV: The International Society for Extracellular Vesicles.
2. EU ME-HaD: the European Network on Microvesicles and Exosomes in Health and Disease.
3. Vesiclepedia.
4. ExoCarta created in 2009 as an open access resource for compiling proteins and RNAs identified in exosomes (referenced in ‘Biochemistry of Exosomes’).

The present review, hence, explores the recent developments in the exosome technology more specifically to their use in breast cancer diagnosis and drug delivery. The review starts with the biogenesis of exosomes and further discusses the various exosomal approaches that have been recently investigated in this domain. Selection of different drug loading techniques either before or after the isolation of exosomes has been discussed in detail. The review also includes in-depth discussion about the concept of exosome surface functionalization by different targeting ligands and its application for obtaining effective diagnosis and treatment for breast cancer. Ongoing clinical trials, in the subject area, have also been included to gain a better understanding of the current status of the exosome technology. Overall, the prime target of the current manuscript is to provide the state-of-the-art involved in the field of exosomes and their advancements in the diagnosis and drug delivery in breast cancer.

## 2. Biogenesis of Exosomes

**Figure 3:**
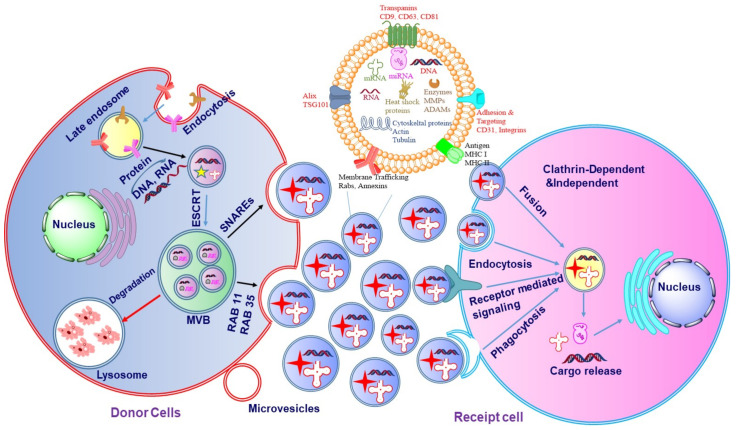
The mechanism of exosome biogenesis. The biogenesis of exosomes is initiated with endocytosis which includes cell membrane inward budding and enclosing of biologically active cargos, which further leads to the development of the endosome. The enclosed cargos are then categorized into smaller vesicles which bud from the perimeter membrane into endosome lumen forming multivesicular bodies (MVBs). Further, MVBs either merge with the lysosome for degeneration or with the inner plasma membrane through RAB or soluble N-ethylmaleimide-sensitive factor attachment protein receptors (SNAREs) to release exosomes.

The biogenesis of exosomes may be divided into three major stages (Figure 3): (i) formation of the endocytic vesicle, (ii) formation of multivesicular bodies (MVBs) which comprise intraluminal vesicles (ILVs), and (iii) the fusion of these MVBs with the plasma membrane [11,12,13]. The exosomes can be secreted both in a healthy state and disease state by a wide array of cell types [14]. During the process of biogenesis, the endocytic vesicle formed from the plasma membrane is first developed into an early endosome and then into late endosome. The limiting membranes of the late endosomes undergo invagination to produce ILVs within the lumen of organelles [15]. The assembling of ILVs into late endosomes leads to the formation of MVBs. The formation of endocyclic vesicles is controlled by specific proteins that belong to the Rab family. The formation of endosomal vesicles occurs via two pathways namely, endosomal sorting complexes required for the transport (ESCRT) dependent pathway and ESCRT-independent pathway. The Rab family proteins include four ESCRT proteins namely ESCRT-0, ESCRT-I, ESCRT-II, ESCRT-III. Apart from the Rab proteins, the other two proteins associated with the transition of endosomes to exosomes include TSG101 and ALIX [16,17,18]. Such a process needs ubiquitination of the cytosolic tail of the endocytosed receptors.

TSG101 belongs to the ESCRT-I protein and frames a complex with the ubiquitinated proteins that activate the ESCRT-II protein and promote the oligomerization and formation of the ESCRT-III complex. This complex then gets involved in the sequestration of MVB proteins and the enrollment of the de-ubiquitination catalyst to eliminate the ubiquitin from the load proteins before arranging them into ILVs. In the last stage of exosome formation, the ESCRT-III complex is degraded by vacuolar protein sorting associate protein 4 (VPS4) and ATPase [19,20]. The schematic diagram of the biogenesis of exosomes is represented in Figure 3. In ESCRT-independent biogenesis of exosomes, ceramide plays an important role which is synthesized by sphingomylinase enzyme [21]. Inhibition of this sphingomyelinase enzyme reduces the secretion of exosomes in Oli-neu cells [12].

Some recent studies have shown that inhibition of these complex proteins (ESCRT, TSG101, and ALIX) resulted in a decrease in exosomal secretion. Four separate investigations have shown that inhibiting Hrs numbers of ESCRT-0 resulted in a decrease in exosomal secretion in different cell types which includes cervical cancer cells (HeLa)—a lineage of cancer human epithelial cells, human embryonic kidney cell line 293 (HEK293), mouse dendritic cells, squamous carcinoma cell line [22,23,24]. The in vivo exosomal secretions are also decreased by the presence of certain drugs such as calpeptin, manumycin, imipramine, etc. Calpeptin is a calcium-dependent neutral cytosolic cysteine protease that inhibits calpains which plays a role in the production of multivesicular bodies [25]. Manumycine is an anti-bacterial agent which inhibits the small GTPases Ras enzyme in F11, mouse neuroblastoma hybrid cells, and rat dorsal root ganglion cells [26], and imipramine is a tri-cyclic anti-depressant which inhibits the acid sphingomyelinase (aSMase) both in vitro and in vivo [27,28]. Depletion of TSG101 also reduces the secretion of exosomes from immortalized retinal pigment epithelial cells 1 (RPE1) [22]. 

## 3. Isolation of Exosomes

Several approaches have been reported in the literature for the isolation of EVs, *viz.,* (1) ultra-centrifugation, (2) size-exclusion chromatography, (3) precipitation, (4) ultra-filtration, and (5) immunoaffinity. Every method has its benefits and drawbacks, however, based upon the exosome source and purpose, the method can be adopted for the isolation of exosomes.

### 3.1. Centrifugation

There are two types of centrifugation methods used for the isolation of exosomes: (1) differential ultra-centrifugation, separation based on the size of the different components and (2) density gradient ultra-centrifugation based on their densities.

#### 3.1.1. Differential Ultra-Centrifugation

In differential ultracentrifugation, exosomes are isolated by serial centrifugation at different times and speeds. Johnstone et al., 1992 first used the differential ultracentrifugation method to separate exosomes from the tissue culture medium [29], which was further upgraded by Thery et al., 2006 [30]. According to Thery et al., cell culture supernatant underwent serial centrifugations at 300× *g* and 2000× *g* for each cycle of 10 min, and 10,000× *g* for 30 min to remove the live cells, dead cells, and cell debris, respectively. The final supernatant obtained was ultra-centrifuged at 100,000× *g* for 70 min to obtain the pellets containing exosomes and proteins which were washed with PBS to procure pure exosomes.

Although, various research groups have adopted ultracentrifugation-based exosomes isolation, however, isolation via differential centrifugation method does not yield a large number of pure exosomes which limits its utilization. Therefore, it is advised to either optimize the existing technique or we need to find an alternative method of isolation that will overcome the problems associated with the differential ultracentrifugation method. This may include the optimization of different process variables (number of steps with variable speeds) for obtaining the pure population of exosomes.

#### 3.1.2. Density Gradient Ultra-Centrifugation

In the density gradient ultracentrifugation (DG centrifugation) method, similar steps are being followed as the differential ultracentrifugation method except for the last stage where the separation/purification is completed in high-density sugar solution or iodixanol which leads to the collection of pure exosomes at the middle layers of the test tube. 

Yamada et al., 2012, isolated exosomes from bovine milk via ultra-centrifugation, the whey from 100 mL of milk was ultra-centrifuged at 100,000× *g* for 60 min, after which the pellets were collected and re-suspended in 1 mL of fresh chilled PBS. The EVs in suspension were segmented on a linear sucrose density gradient (10–40%) solution (9 mL) and were again ultra-centrifuged at 200,000× *g* for 18 h. Fractions (0.9 mL) were collected from the top of the centrifuge tube and categorized from 1 to 10, where the fraction numbered 7 was confirmed as exosomes by using the Western blot analysis. Fractions numbered 7–9 were diluted with chilled PBS and again ultra-centrifuged at 100,000× *g* for 60 min to obtain pure exosomes which were further characterized by electron microscope [31]. However, DG centrifugation is a more convenient method to isolate exosomes at high yield and purity, although it consumes more time and is a high equipment-dependent method which limits its usefulness.

### 3.2. Chromatography-Based Isolation Method

In this method, exosomes are separated based on size differences between the EVs in biological samples by using size-exclusion chromatography (SEC) which is also known as gel filtration or molecular sieve chromatography. The source material of the EVs is loaded on the column packed with Sepharose©, Sephacryl©, or BioGel P, as a stationary phase. Thus, the elution is based upon their size in the order of decreasing molecular weight [32]. The commercially available columns which are employed to isolate the exosomes include qEV separation columns, EV Second purification columns, Exo Cap™, and Exo-spin. Compared to ultracentrifugation and ultrafiltration, the chromatography-based isolation method is quick and of relatively low cost. Additionally, the isolated fractions are uniform in size and their biological characteristics remain significantly unaffected by this method. However, there are some chances where exosomal fractions may get contaminated with other particles of the same size which may compromise the purity of exosomes. Nevertheless, owing to its time-consuming process, the SEC method limits its use in treatment and research. 

A comparative study was performed by Gamez-Valero et al., 2016 [33], where the exosomes were isolated from plasma by SEC using Sepharose^®^ CL-2B as a stationary column, followed by precipitation with PEG, and the PROSPR method. Exosomes from the SEC method may contain a lower amount of contaminated proteins with the highest quantity of exosomal biomarkers such as CD9, CD63, CD81, and CD5L [33].

### 3.3. Ultrafiltration-Based Isolation

**Figure 4:**
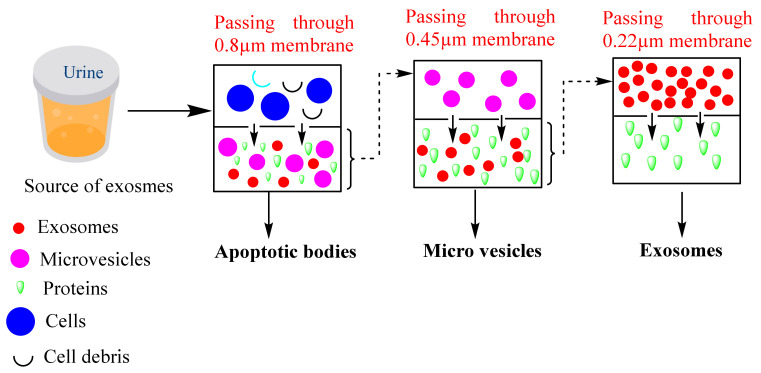
Common steps followed during the separation of exosomes via ultrafiltration. Here, the samples are passed through multiple membranes of different pore sizes such as 0.8, 0.45, 0.22 µ which result in the concentration of the sample after every pass through the filters of different pore sizes.

Ultrafiltration-based isolation of exosomes depends upon the separation of the different classes of EVs such as apoptotic bodies, microvesicles, exosomes, and other contamination proteins based on their different sizes. The filtration-based isolation method is classified into three types, *viz.,* ultrafiltration, hydrostatic dialysis, and gel filtration. In the ultrafiltration method, the ultrafiltration membranes are used to isolate exosomes that are available in different pore sizes such as 0.8, 0.45, 0.22, and 0.1 µm capable of retaining the particles with diameters of 800, 450, 220, and 100 nm, respectively. Larger particles such as apoptotic bodies are separated first followed by microvesicles and exosomes [34]. A schematic diagram of the filtration-based method is represented in Figure 4. Based on this method, Cheruvanky et al., 2007 have isolated exosomes from urine. They utilized ultrafiltration cells with nanomembranes which exhausted the protein with a molecular weight greater than 100 kDa, then centrifugation at 3000× *g*. Such a method does not require any further ultracentrifugation [35]. Recently a comparative study showed that the yield of the exosomes is greater in the ultrafiltration method compared to the ultracentrifugation method. Moreover, in the ultrafiltration method, the size of the exosomes is below 100 nm which indicates that the ultrafiltration method is most suitable for isolating exosomes from large sample sizes [36].

### 3.4. Precipitation-Based Isolation

The precipitation-based isolation method is widely used for the isolation of exosomes. As per an earlier report, precipitation-based strategies are utilized particularly for biologics having a low initial volume. It was observed that 84% of scientists utilize the precipitation method for investigating the encapsulation of RNA in EVs [37]. In the precipitation method, polymers such as polyethylene glycol (PEG) [38], salt solutions such as sodium acetate [39], organic salts, and a charge-based moiety such as protamine are used. The exosomes are first incubated with a precipitating agent for 1–24 h depending on the nature and concentration of the precipitating agent, followed by low-speed centrifugation [38]. With the emergence of the requirement for exosomes’ isolation, several biotech companies are developing commercial kits (Table 2) for the precipitation-based isolation of exosomes. In the protein organic solvent precipitation (PROSPR) method, solvents such as acetone, chloroform, glacial acetic acid, and trichloroacetic acid are used to precipitate the solubilizing proteins. Somiya et al., 2018 isolated exosomes from cow milk based on the PROSPR method [40]. In the charge-based precipitation technique, protamine sulfate is used for separation. Protamine sulfate is a positively charged molecule that interacts with the negatively charged EVs and forms a precipitate that is separated by low-speed multiple centrifugations [41]. The precipitation-based isolation method is the most promising isolation method for clinical use due to the requirement of lower sample quantity, less time consuming, and absence of exosomal damage. However, the precipitation method is facing the problems of contamination with other exosomal proteins such as albumin and PEG which could lead to the toxicity [42]. Nevertheless, to minimize the contamination, pre-filtration through 0.22 um or post-precipitation–purification steps such as filtration and centrifugation were performed to reduce the contamination with impurities [43].

### 3.5. Immunoaffinity-Based Isolation

Immunoaffinity is a separation and purification method based on certain interactions between antibodies and antigens to isolate the required component from a mixture. This is the most promising method for the isolation of specific exosomes. The commonly used antibodies in immunoaffinity-based isolation are monoclonal antibodies against specific biomarkers: CD9, CD63, CD80, and TSG101 for exosomes; integrins and CD40 for microvesicles, and annexin V for apoptotic bodies. These antibodies are fixed on different types of materials such as magnetic beads or microfluidic devices. Nakai et al., 2016 isolated exosomes from mouse peritoneal macrophages cells. For this, Tim4-FC protein was used to bind with phosphatidylserine present on the surface of the exosomes which were then captured by using magnetic beads [44]. However, immunoaffinity-based isolation is not suitable for isolating a large number of exosomes. Moreover, the high cost and low yield of exosomes limit their usefulness.

Patel et al., 2019, and Tang et al., 2017, isolated the exosomes using a different method and observed that exosomes isolated via the UC method showed higher purity as determined by particle and protein ratio, and exosomal biomarker (CD9) as compared to SEC [45,46]. However, further studies are indispensable to determine the purity of exosomes from different isolation methods. 

The advantages and disadvantages of different isolation methods are discussed in (Table 3).

## 4. Drug Loading

**Figure 5:**
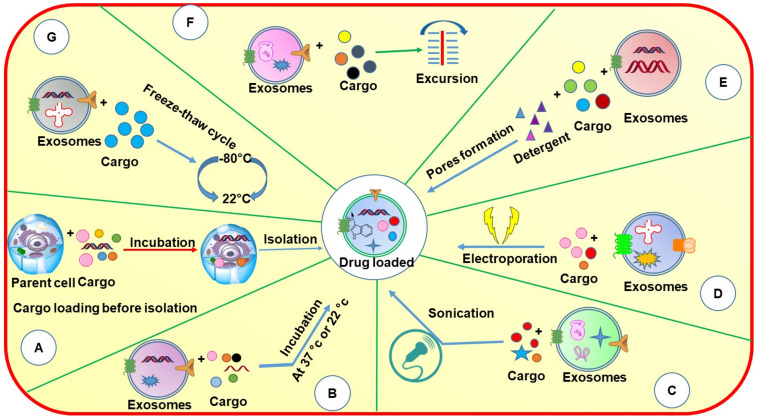
Schematic diagram showing different methods of loading cargos into exosomes through pre-loading and post-loading strategies: (**A**) incubation with parent cell; (**B**) incubation; (**C**) sonication; (**D**) electroporation; (**E**) detergent method; (**F**) excursion; (**G**) freeze–thaw cycle.

Exosomes can be used as nanocargos that are capable of loading different small and large molecules. They can load drugs either before or after isolation depending on the application (Figure 5). Each of these loading methods has its pros and cons (Table 4).

Drug loading before isolation of exosomes can be performed in two ways: (1) incubation of parent cells with a drug and (2) by gene editing. In the incubation method of drug loading, the compounds are directly mixed with the cell culture medium, where the drugs get internalized into cells and are loaded into EVs via an endogenous mechanism. Tang et al., 2012 incubated mouse hepatocarcinoma tumor cells (H22), Raw264.7 cells, human ovarian cancer cells (A2780, B16, HL60, and EL4) with four different drugs namely methotrexate (MTX), doxorubicin (DOX), cisplatin, and hydroxyl camptothecin for 12 h. MTX was incubated with H22 cell lines at different concentrations (1–10 μg mL^−1^) and then ultracentrifuged to obtain microparticles which were again incubated with the same cell lines for 48–72 h at different concentrations. It was observed that both the high concentration and low concentration derived microparticles induced cell death, but the high dose derived microparticles resulted in microparticles packaged with a large amount of drugs, as compared to low dose derived microparticles [47]. In another study, Pascucci and colleagues incubated paclitaxel (PTX) with SR4987 cells (bone marrow mesenchymal stromal cells) at low concentrations (100 µg mL^−1^) for 24 h. After 48 h, exosomes were collected from the culture medium by differential centrifugation. The isolated PTX-exosomes showed significant anti-proliferative activity against CFPAC-1 cell lines (human pancreatic adenocarcinoma cells) [48]. Interestingly, the exosomes devoid of paclitaxel also showed a minor anti-proliferative activity, due to the presence of some nucleic acids and proteins that may change the tumor environment.

### 4.1. Drug Loading after Isolation of Exosomes

Several methods have been used for drug loading after the isolation of exosomes. The drug loading can be achieved by incubation, sonication, detergent treatment, electroporation, extrusion, and freeze–thaw cycle to load the drugs following the isolation of exosomes from the respective biological source. All the loading techniques mentioned above are adopted from the field of liposomes by considering exosomes as a cousin of liposomes with higher stability and better biocompatibility. Except incubation, other loading techniques are based upon the assumption that the exosome lipid bilayer will rearrange in its original form similar to liposomes following the application and then removal of stress. Although this assumption may be correct, the effect on the biological activity of integral proteins and their rearrangement in the original position needs to be explored further. While every method has its advantages and disadvantages, incubation may be considered the simplest or safest method of drug loading which is not supposed to change the structures and the different components.

#### 4.1.1. Incubation

Incubation is the simplest technique to load drugs into/onto EVs. In this method, the drug solution is simply mixed with isolated exosomes and incubated at different temperatures (body temperature and room temperature) and in the presence of a lower percentage of organic solvents which is supposed to allow the drug diffusion into the exosomes or hydrophobic interaction onto the exosomal surface protein. The level of drug diffusion is again dependent upon the physicochemical properties of the drug as the hydrophobic drugs can easily interact with the lipid layer of the exosome membrane and hence can permeate easily [49]. Yang et al., 2015, compared the entrapment efficiency of two exosomal formulations loaded with paclitaxel and doxorubicin, respectively. Both the drugs were incubated with exosomes at body temperature for 2 h and it was found that doxorubicin showed slightly increased entrapment efficiency in comparison with paclitaxel [50]. We reported the loading of milk-derived exosomes with chemotherapeutic drug PTX by incubation method and observed around 8% of practical loading. Interestingly, the exosomal drug exhibited excellent stability in the presence of simulated-gastrointestinal fluids, and during the low-temperature storage. The exosomal paclitaxel delivered orally showed significant inhibition of lung tumor xenografts and did not elicit any systemic and immunotoxicity compared to *i.v* paclitaxel [51]. Similarly, by using the incubation method, various drugs, and nucleotides, *viz.,* curcumin, celastrol, anthocyanidins, siRNA, and withaferin A were loaded into/onto milk-derived exosomes and delivered successfully into the animals to treat various types of cancers including breast, lung, ovarian, etc. [52,53,54,55,56]. The incubation method is simple and inexpensive in comparison with other techniques, however, not efficient in terms of loading a large number of hydrophilic drugs, nucleic acids, and proteins.

#### 4.1.2. Sonication

Sonication is a physical method in which mechanical shear force is applied by using a probe sonicator which decreases the exosome’s membrane integrity and allows the entry of drugs and other proteins into the exosomes. After which, the membrane is recovered by incubating the exosomes for 30–60 min at 37 °C. This method can be used to load a relatively large amount of drugs into exosomes.

Kim et al., 2016 formulated paclitaxel and doxorubicin-loaded exosomes using three different methods, including incubation at room temperature, electroporation, and sonication. It was found that the sonication method provided higher entrapment efficiency of PTX in the exosomes followed by electroporation and incubation at room temperature. Hence, it was indicated that the sonication method is the best method to load large amounts of drugs into exosomes [57]. However, exosomes may lose integrity due to high mechanical shear. Although, the sonication method was observed to provide the maximum drug loading to exosomes and could be used for both small and large molecules, the effect of sonication on the exosomal surface biomarkers/proteins and their integrity need to be confirmed.

#### 4.1.3. Electroporation 

Electroporation is a well-reported method for the loading of hydrophilic drugs, siRNA, and miRNA into the exosomes as these macromolecules are larger and cannot be loaded to the exosomes by simple diffusion. In this method, exosomes are suspended in an electroporation buffer and the electric field is applied which results in the formation of small pores in the exosomal membrane leading to entry/loading of nucleotides into the exosomes. Although there are several successful reports about this method, RNA precipitation and aggregation of exosomes are also reported in some of the studies [58]. Hood et al., 2014 proposed that the exosomal membrane aggregation and RNA precipitation can be reduced by using membrane stabilizers such as trehalose pulse media due to their increased colloidal activity [59]. However, some scientists do not prefer the use of stabilizers as they might get attached to the membrane of exosomes.

The drug entrapment efficiency in the electroporation method depends on parameters, *viz.,* voltage, capacity, and pulse duration. Generally, in all electroporation methods, a voltage of 350–400 V has been used. Aqil et al., (2019), showed that electroporation could only load about 5% siRNA while chemical transfection resulted in about 30% loading [56].

#### 4.1.4. Extrusion, Detergent Treatment, and Freeze–Thaw Cycle

In the extrusion method, the exosomes are first mixed with drug solution and loaded into a syringe-based lipid extruder. The solution is then allowed to extrude through membranes of 100–400 nm pore size at controlled temperature. While passing through the membrane, the exosomal membrane gets disrupted which then allows their vigorous mixing with the drug solutions, resulting in effective drug loading into the exosomes. However, due to the usage of mechanical forces, the properties of the exosomal membrane such as zeta potential and protein structures can undergo certain changes. Fuhrmann et al., 2014 demonstrated that loading of porphyrin in MDA-MB-231 breast cancer cell line-derived exosomes by using extrusion method (sample was extruded 31 times) have shown some significant phototoxicity, whereas porphyrin loaded into liposomes did not show any significant phototoxic effect, due to the lack of encapsulation [60]. 

In the detergent treatment method, some of the detergents such as saponin have been used, which form the complex with cholesterol in the cell membrane resulting in pore formation and increased membrane permeation [61]. The size of the membrane pores is approximately 100 Å, which promotes the incorporation of therapeutic agents into exosomes.

In the freeze–thaw cycle method, the drugs are first incubated with exosomes at 37 °C for a specific time, after which the mixture quickly freezes at −80 or −160 °C (in presence of liquid nitrogen) followed by defrosting at room temperature. This cycle of freezing and unfreezing is repeated at least three times [62]. Although this method may result in a good loading capacity, stress-induced aggregation of exosomes may pose a problem of increase in the size of drug-loaded exosomes. Haney et al., 2015 proposed that the loading of drugs into exosomes by freeze–thaw cycle is significantly lower than the sonication and incubation methods [63]. Sato et al., 2015 used the freeze-cycle method to fuse the exosomes with liposomes to mimic the actions of liposomes [64]. However, the use of saponin as a loading material is limited because of the hematolytic nature and possible toxicity in vivo.

## 5. Applications of Exosomes in Breast Cancer

### 5.1. Biomarkers

Exosomes play a crucial role in intracellular communication by directly binding with surface receptors or transferring their contents to another cell [65]. The presence of exosomal RNA was embroiled as proof for the horizontal transfer of genetic information between different cell types [66]. Valenti et al., 2006 and Whiteside et al., 2013 demonstrated that exosomes can transfer cellular RNA as well as miRNA which indicated that the tumor exosomes showed some functional effects including the suppression of the mRNA which codes for signal transduction components within the T-cell [67,68]. Exosomes secreted from cancer cells show a higher number of RNA due to the higher production of mRNA and miRNA and thus the miRNA may reflect the parental tumor signature. As a result, miRNA expression profiling could be used as a biomarker in diseases, including some cancers specifically in those cancers that lack specific molecular biomarkers.

Baroni et al., 2016 observed that cancer cell-secreted miR-9 could be carried by the exosomes to the healthy fibroblast cells, and thus the uptake of miR-9 can convert the healthy fibroblast into cancer-associated cells, which further lead to increased cell mortality of breast cancer cells [69]. Recently, Shen, et al., 2021 observed elevated levels of exosomal miR-7641 by using qRT-PCR and microarray in the plasma of patients suffering from breast cancer, which is considered a potential diagnostic marker in breast cancer. They further demonstrated that the elevated levels of exosomal miR-7641 could promote tumor growth in vivo [70]. In another study, Hannafon et al., 2016 observed an elevated level of exosomal miR-21 and miR-1246 in plasma of breast cancer patients. This identification indicated their potential as a biomarker in breast cancer [71]. The higher levels of miRNA in breast cancer patients also represented chemo resistance. To confirm this, Liu et al., 2021 isolated exosomes from MCF-7 cells which contained miR-9-5p. It was observed that MCF-7/tamoxifen caused miR-9-5p inhibited apoptosis in cancer cells and increased the cell resistance to tamoxifen treatment [72].

Recently, Hirschfeld and co-workers performed a comparative study in 69 breast cancer patients vs. healthy humans. From the study, four highly expressed urine biomarkers (miR-424, miR-423, miR-660, and let7-i) were identified in breast cancer patients, which represented 100% specificity and 98.6% sensitivity [73]. The high levels of long non-codingRNAs (IncRNA) and non-protein coding RNA (DANCR) are considered potential biomarkers in breast cancer. Shi et al., 2022 has discovered an elevated level of both IncRNA and DANCR in breast cancer patients as compared to healthy patients. However, this study was performed in a small group of patients (120 breast cancer patients). An analysis in a large group of BC patients is needed to further confirm the role of serum level of exo-IncRNA and DANCR [74]. Apart from the biological investigation Liu et al., 2021 performed a comprehensive bioinformatics analysis to discover the highly expressed miRNA by gene expression omnibus. It was observed that the exosomes, tissues, and cells showed upregulated levels of miR-21-5p. Furthermore, from the characteristic analysis, it was also confirmed that miR-21-5p could be effectively differentiated in BC patients and healthy people with 87.7 sensitivity and 93.3% specificity [75].

Exosomal miRNA was also identified in serum, plasma of breast cancer patients, and in vitro cell culture that potentially helped in early detection of breast cancer. Several potential exosomal miRNAs and proteins for breast cancer are summarized in Table 5. Nevertheless, the physiognomies of circulating tumor cells and cell-free DNA (cf-DNA) related to cancer cell DNA are still unclear as compared to exosomal tumor biopsies. Additionally, cf-DNAs carry mutations distinctively of the consistent tumor cells. However, the clearance of circulating DNA is usually observed in the kidney or liver, indicating the steadiness and pathogenicity of circulating DNA. Therefore, further investigation is indispensable to discover the most targeted and promising set of miRNAs which is highly correlated with solid tumor RNA.

### 5.2. Drug Delivery in Breast Cancer

Exosomal drug delivery gained a lot of interest over the past decades because of the various advantages, *viz.,* biocompatibility, low toxicity, high stability, long circulating half-life, and tissue targetability. In addition to being natural in origin, they can also be used to deliver both hydrophilic and hydrophobic small molecules and macromolecules such as nucleic acids and proteins.

#### 5.2.1. Exosomal Delivery of Small Molecules

Exosomes have been used as a drug delivery system for the delivery of several small molecules of both hydrophilic and hydrophobic nature. In several cases, exosomal delivery leads to a higher accumulation of drugs at targeted sites. Exosomal delivery also improves the stability of small molecules and increases their stay in systemic circulation which further improves the pharmacokinetics and thus the therapeutic efficacy. In our previous studies, we were able to deliver different natural and synthetic compounds such as withaferin A, anthocyanidins, curcumin, paclitaxel, and docetaxel using cow milk-derived exosomes [51,52,53,54,55]. The drug loading was completed using simple incubation which also showed a sustained release profile over time. It was also observed that the exosomes loaded with withaferin A and paclitaxel exhibited lower IC_50_ values as compared to free drugs in MDA-MB-231 breast cancer cells. In addition, exosomes also demonstrated improved anti-tumor activity of tested compounds in vivo in tumor-bearing mice. Exosomes loaded with withaferin A exhibited a significantly higher inhibitory effect on tumors as compared to free withaferin A [52]. In our previous work, we have also successfully formulated paclitaxel-loaded exosomes derived from bovine milk to treat lung cancer. The paclitaxel-loaded exosomes showed significantly higher tumor inhibition in comparison with free paclitaxel in a xenograft model. Additionally, in comparison with free paclitaxel, the paclitaxel-loaded exosomes exhibited remarkably lower systemic and immunogenic toxicities [51]. Toffoli et al., 2015 delivered doxorubicin by using exosomes that were derived from MDA-MB-231 and HCT-116 cell lines. However, in both in vitro and in vivo studies, the exosomal doxorubicin showed the same effect as free doxorubicin but the cardiac toxicity was found to be reduced by exosomal doxorubicin compared to free doxorubicin [88]. On other hand, exosomal delivery of chemotherapeutic agents has improved the ferroptosis in TNBC [89]. Yu et al., 2019 successfully delivered erastin loaded exosomes into MDA-MB-231 cells, however, the surface-modified exosomes with folate have shown more cellular uptake as compared to unmodified erastin loaded exosomes [89]. The delivery of chemotherapeutics and other drugs via exosomes for the treatment of breast cancer is summarized in Table 6.

#### 5.2.2. Exosomal Delivery of Biologics

“Mother Nature” has beautifully loaded a variety of miRNA and other biologics into the exosomes and they are believed to deliver this cargo to the recipient cells. This information ignited the scientific community to explore if the exogenous genetic material/biologics can also be loaded and successfully delivered to the cells to have the desired therapeutic efficacy in different disease conditions. Many successful reports came into the public domain which confirmed the successful loading and then gene silencing by using milk exosomes from different sources, including bovine raw milk. Among the different genetic materials, siRNA is a class of double stranded RNAs that could regulate the expression of specific genes by causing mRNA excision or restraining mRNA translation. However, siRNAs are not therapeutically stable and tend to degrade quickly while in the systemic circulation, which makes it very difficult to deliver these siRNAs to the target cell. We have shown that siKRAS loaded into milk-derived exosomes was protected from enzymatic degradation and able to inhibit the tumor growth in lung tumor xenografts [56]. Alvarez-Erviti et al., 2011 first delivered siRNA by using exosomes as a delivery vehicle. In this study, they used the mouse dendritic cell-derived exosomes and loaded them with siRNA of the BACE1 gene [105]. Recently Munagala et al., 2021 successfully delivered wild-type p^53^ p^DNA^ to mice and H1299 cells. Interestingly they observed high expression of the P^53^ gene in vitro [106]. On the other hand, McAndrews et al., 2021 delivered CRISPR/Cas9 to target mutant Kras^G12D^ to suppress the proliferation and inhibit the tumor growth [107]. Similarly, Sheykhhasan et al., 2021 efficiently delivered miR-145 into breast cancer cells via exosomes to explore the role of miR-145 in metastasis and apoptosis. In this study, the mesenchymal stem cell-derived exosomes were used for loading miR-145. The exosomes showed enhanced inhibition of metastasis and increased apoptosis in breast cancer cells [108]. In a recent study, Xu et al., 2021 successfully delivered antisense oligonucleotide via exosomes to analyze their cellular uptake study in MDA-MB-231 and HepG2 cell lines. Antisense oligonucleotide-loaded exosomes showed enhanced HepG2 cell uptake as compared to the free oligonucleotide. It is worth mentioning that exosomes also possess an ability to bypass the blood–brain barrier (BBB), hence, exosomes can also be used to deliver entrapped therapeutic moieties to the CNS which may be a good opportunity to target the breast cancer metastasized to the brain [109]. In another study, Lee et al., 2011 overexpressed the major histocompatibility complex (MHC) class II protein in murine melanoma cell lines (B16F1) by transduction of the CIITA (Class II transactivator) gene. The exosomes were then isolated from the MHC-II overexpressed B16F1 cell containing a large amount of MHC-II and tumor antigen TRP2. It was observed that this Exo-CIITA exhibited a significantly improved anti-tumor immune response through solenocyte proliferation and IL-2 secretion [110]. The exosomal delivery of nucleic acid and proteins for breast cancer therapy is summarized in the Table 6.

#### 5.2.3. Exosome Modification for Targeted Drug Delivery for Breast Cancer

**Figure 6:**
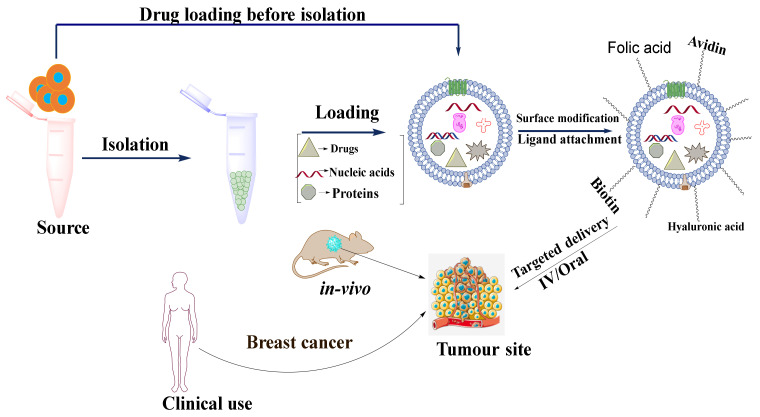
Schematic diagram showing the application of exosomes as a nanocarrier for targeted drug delivery in cancer.

One of the drawbacks of exosomal delivery is that the naturally secreted exosomes in the body can freely move across the extracellular space and biofluids by free diffusion and are randomly internalized into accepter cells. For observing the biodistribution of exosomes, Wiklander et al., 2015 labeled exosomes with DiR dye. It was observed that the exosomes were accumulated in the liver, spleen, kidney, pancreas, and other organs administrated by tail vein which indicated uncontrolled biodistribution of exosomes in vivo [111]. Thus, it was inferred that the delivery of the exosome-loaded drugs to specific targets may require some surface modifications. The modifications are performed in three ways: (1) ligand-receptor binding-based targeted delivery, (2) pH gradient/surface charge-driven targeted delivery, and (3) magnetism-guided targeted delivery. A schematic diagram of targeted drug delivery in cancer therapy is shown in Figure 6.

##### Ligand-Receptor Binding-Based Targeted Delivery

The ligand-receptor binding-based targeted delivery is the most widely reported targeting strategy in which a ligand is attached over the surface of exosomes that recognizes its specific receptor overexpressed on the targeted site or cell. Ligand-receptor binding-based targeted delivery has two types, namely (a) transfection-based ligand overexpression and (b) chemical assembling of ligand on the exosomal surface.

##### Transfection-Based Ligand Overexpression

Cancer cells are characterized by uncontrolled proliferation, migration, abnormal elevation of cellular metabolisms, and overexpression of certain kinds of proteins and receptors such as epidermal growth receptor factors (EGRF) [112]. Ohno et al., 2012 modified MDA-MB-231 cell lines to express the transmembrane domain (TD) of platelet-derived growth factor receptor (PDGFR) by fusing with the GE11 peptide. PDGFR-TD enhances the expression of GE11 peptide on the surface of the exosome which binds specially to overexpressed EGFR. Afterward, Luciferase-expressing HCC70 cells were transplanted into the mammary fat pads of RAG2–/– mice and the GE11 expressed exosomes loaded with let-7a and labeled with DiR dye were administrated systemically to the RAG2–/– mice. After 24 h of incubation, a large number of accumulated exosomes were observed in the spleen and liver [113]. Based on this study, various other studies were performed to target the exosomes over other overexpressed moieties such as Lamp2b and phosphatidylserine [114,115].

##### Chemical Assembling of Ligand on Exosomal Surface

Chemical modification is a method that directly places ligands either on the membrane of donor cells or on the surface of the exosomes by using chemical interactions. In our previous studies we labeled the exosome with tumor-targeting ligand, folic acid, and then loaded it with plant therapeutic, withaferin A. The surface functionalization with folic acid showed enhanced inhibition of tumor growth as compared to the exosomal withaferin A without surface functionalization [52]. Recently for enhanced tumor targeting, we further modified the surface of the exosomes with activated folic acid via a covalent bond in which the folic acid was simply activated by EDC/NHS chemistry and supposed to form covalent bonds with the amine groups present over the exosomal surface. Functionalization by using covalent bond was supposed to be stable until the functionalized exosomes reached the target site. In line with our hypothesis, it was observed that the folic acid-modified exosomes showed a significant reduction in tumor size as well as immunotoxicity as compared to non-targeted exosomes and available marketed paclitaxel formulation [116]. Wang et al., 2017 first labeled the donor cell membrane chemically with biotin and then labeled exosomes with avidin. Exosomes were then isolated by microfluidic devices and loaded with doxorubicin. These dual targets showed strong targeting abilities towards the liver cancer model [117]. Recently, Li et al., 2020 delivered hyaluronic acid coupled doxorubicin exosomes to CD-44 overexpressed tumor cells, as hyaluronic acid acts as a specific ligand of CD-44 receptor [118].

##### pH Gradient/Surface Charge-Driven Targeted Delivery

The specific physicochemical properties of different tissues and cells play an important role in targeting the exosomes. For example, increased intracellular glycolysis and lactate production creates an acidic environment around the tumors which makes the pH-responsive medication prominent in targeting the tumors. Kim et al., 2018 successfully delivered doxorubicin loaded in i-motif-modified (i-motif-bio) exosomes by using a pH gradient. Interestingly, da-i-motif-bio was efficiently released in acidic pH within one hour [119]. On the other hand, Zhang et al., 2020 prepared doxorubicin-loaded exosomes which were further conjugated with a moiety having a pH cleavage bond that undergoes cleavage in acidic conditions. Moreover, endoperoxides and chlorin e6 (Ce6) were also loaded where the endoperoxides undergo thermal cycloreversion and release singlet oxygen that kills the cancer cells in squamous cell carcinoma [120].

##### Magnetism-Guided Targeted Delivery

Apart from techniques that use the physicochemical and biological characteristics of specific tissue or cells, targeted drug delivery can be accomplished with the help of some external magnetic forces. Qi et al., 2016 developed a dual-functional exosome-based superparamagnetic nanoparticle cluster to obtain a targeted drug delivery vehicle for cancer therapy. Engineered exosomes exhibit a strong response to an external magnetic field, which enables the exosomes to become separated from the blood and target the cancer cells. In vivo examinations further showed that the exosomes have an attractive capacity to deliver doxorubicin to malignant cells to suppress their further progression [121].

Exosomes served as a natural and novel drug delivery system that can deliver potent anticancer drugs to target tumors. Nevertheless, administering exosomes as a drug delivery system may present a certain disadvantage, because exosomes loaded with protein, miRNA, and nucleic acids may provoke the transformation of healthy cells to cancer-associated cells, thereby leading to tumor development. Therefore, further studies should be concentrating on exact loading mechanisms to exosomes, discovering novel loading methods and isolation methods to produce a large number of exosomes for industrial applicability, and novel technology for delivering exosomes to treat various diseases, including breast cancer, without causing any pharmacological adverse effects and toxic effects caused by endogenous and exogenous exosomes.

## 6. Challenges of Exosomes in Drug Delivery and Biomarker

The exosomes do possess certain challenges that need to be addressed. The first challenge faced by the exosomes is the lack of an appropriate standardization technique that should be employed for the isolation and purification of exosomes. The general method used for isolation is multi-step ultracentrifugation which is considered the “gold standard method” and at the same time, there arises a risk where the exosomes obtained might get contaminated with other types of EVs. Absence of a precise isolation and purification method leads to a risk of having both exosomal and non-exosomal EVs in the group of obtained exosomes. The existence of non-exosomal EVs may further compromise the therapeutic efficacy of the exosomal EVs. Secondly, for employing exosomes for cancer therapy, one should prevent sourcing exosomes from cancer cells because they might contain oncogenes that will facilitate cancer progression. Finally, the extraction of exosomes from cell cultures may display variability in their properties even though they are extracted from the same type of donor cells. It was observed that the cell culture and purification techniques limit the implementation of exosomes as therapeutic vesicles as well as hinder their mass production.

In addition, the exosomes are also used as biomarkers which are also presented with some challenges. Cells produce certain sets of proteins and miRNAs; it was observed that the exosomes also produce or express similar sets of proteins and miRNAs with few distinct cell-specific proteins. Further, because the exosomal populations expressed from single cells are heterogeneous, the content concentrations of the exosomes are expected to reside in a range rather than a set standard. Additionally, the exosomes are isolated from various types of cells, so unless they present some exceedingly specific cargoes, the determination of their tissue of origin would be challenging. To date, there remains a lack of compiled data based on exosomes required for the diagnosis of diseases. Additionally, there is no convenient method in terms of time, sample throughput, quality control, and accuracy or regulated technologies under the clinical level that could be used for detection and analysis of exosomes.

## 7. Current Clinical Trials of Exosomes in Breast Cancer Therapy

Based on several reports, exosomes can be considered one of the most effective delivery vehicles in the treatment of breast cancer. Tremendous scientific efforts and several positive outcomes could force exosomes up to the stage of clinical trials (Table 7), yet, the concept of exosomes is far beyond the clinical reality. The current trials on exosomes are mainly focused to identify potential exosomal biomarkers in different biological fluids, their role in drug resistance, and studying the effect of anti-cancer drugs on metastasis by exosomal genome analysis. An ongoing clinical study on exosomes includes proteomic analysis of cerebrospinal fluid-derived exosomes of 72 breast cancer patients for the diagnosis of leptomeningeal metastatic breast cancer. Similarly, in another clinical study, exosomes were isolated from blood and urine samples of patients suffering from breast cancer to quantify the stress protein (HSP 70), which is considered a solid tumor biomarker. Apart from the exosomes as biomarkers, their application in chemotherapy is also under consideration. For instance, in the case of HER2 type breast cancer, the HER2 receptors are also found to be overexpressed on the surface of exosomes, so in the current run, the scientists diagnose cancer by quantifying the expressed HER2 and HER3 dimers over the isolated exosomes. In a similar context, the effect of pembrolizumab in the tumor microenvironment can also be established by observing the isolated exosomes. Similarly, in an ongoing phase II clinical trial, tumor-derived exosomes were analyzed proteomically to determine the combined effect of pembrolizumab and enobosarm in androgen receptor-positive TNBC. It could be stated that as the exosomes serve as a potential biomarker for early detection of breast cancer and a carrier for providing a therapeutic response to chemotherapy, the identification of any particular biomarker provides a great deal of information in early diagnosis and treatment. Moreover, being non-invasive may provide a breakthrough discovery and a great level of patient compliance.

## 8. Conclusions

Exosomes are nanosized lipid-based small EVs that have been successfully used as a diagnostic tool and delivery vehicle for a variety of bioactive, *viz.,* small molecules, proteins, DNAs, and RNAs such as mRNAs, miRNAs, etc., to the desired site and modulate the cellular communication within the tumor microenvironment. Surface functionalized exosomes further represent a novel approach to have their specific accumulation at the site of interest minimizing off-target effects and effective management of various cancers including breast cancer. Exosomes can be employed for therapeutic advantages by profiling their contents to assist with the diagnosis of breast cancer, evaluating their unique molecular characteristics to observe their distribution locally or distally, and transforming them into a targeted drug delivery system for cancer treatment. Recognition and modification of various cell-derived exosomal contents may provide a platform for novel diagnostic, preventive, and therapeutic strategies with minimally invasive techniques. Despite being a boon in targeted drug delivery systems against breast cancer, exosomes face certain challenges which include the need for standardization parameters for the classification of exosomes, finding the methods for determining the behavioral interaction of tumor microenvironment and exosomes as the tumor microenvironment is associated with the progression of different stages of breast cancer, and establishing their precise role in organ metastasis. Hence, further work in the field of exosomes is warranted to widen our understanding of the role of exosomes in breast cancer along with improved breast cancer treatment.

## Figures and Tables

**Table 1:** Classification of EVs based on their size, biomarker, and biogenesis process.

Type	Size (nm)	Density (g/mL)	Morphology	Protein Markers	Origin	Biogenesis Process	Composition
Exosomes	30–150	1.13–1.19	Cup shaped	CD9, CD63, CD80, TSG 101, Alix	Late endosomes	Inward luminal budding	mRNA, miRNA, DNA, heat shock proteins
Microvesicles	50–1000	1.04–1.07	Heterogeneous	Integrins, selectins, CD40, Flotiline-2.	Plasma membrane	Outward luminal budding	mRNA, miRNA, DNA, Cytoplasmic proteins
Apoptotic bodies	50–5000	1.16–1.28	Heterogeneous	Annexin V positivity, Phosphatidyl serine	After cell death	Cell shrinkage	Cell organelles, DNA, RNA, histone

**Table 2:** Commercially available exosome isolation kits based on the principle of precipitation.

Exosome Isolation Kit	Manufacturers
Exosome isolation kit	Cusabio Technology LLC.
exoEacy maxi kit	QIAGEN.
Total exosome isolation kit	Thermo Fisher
Saliva exosome purification kit	Norgen Biotech corp
Capturem™ Exosome Isolation Kit	Takara
Magcapture™	Fujifilm wako Chemicals
BasicExoRNA™ extraction kit	Bio vision.
ExoQuick plus	SBI
EZB-exo1	EZ bioscience

**Table 3:** Advantages and disadvantages of various isolation methods.

Isolation Method	Advantages	Disadvantages	Size (nm)
Differential UCF	Simple and low cost, most adopted method	Low yield, possible mechanical damage, time-consuming process	75–90
Density gradient UCF	Provide high purity of exosomes	Low yield, time-consuming process, skills required	80–100
Chromatography based	Simple and fast, absence of exosomal damage, useful for biological samples	Contamination with other proteins and PEG	108–130
Ultrafiltration	More yield compared to UC	Low purity	100–170
Precipitation	Less sample requirement	Non-exosomal contamination	120–140
Immunoaffinity	Ultra-pure yield, requires lower sample sizes, simple and fast	Low yield, high cost, not suitable for large quantities	20–110

**Table 4:** Summary of different drug loading methods.

Method	Mechanism	Advantages	Disadvantages
Incubation before isolation	Drugs are internalized into cells and loaded into the vesicles by the endogenous mechanism of cells	Useful for hydrophobic drugs, does not affect the integrity of exosomes	Not useful for hydrophilic drugs, low drug loading capacity
Incubation after isolation	Passive diffusion method	Simple method, useful for hydrophobic drugs, does not affect the integrity of exosomes	Not useful for hydrophilic drugs, low drug loading capacity, cannot load nucleotides
Sonication	The mechanical shear force from the sonicator probe decreases the membrane integrity and forms the pores	A large amount of drugs can be loaded	Can damage the proteins, other components, and integrity
Electroporation	By applying a high voltage electrical charge to create temporary pores on the exosomal membrane	Useful for loading of hydrophilic drugs, siRNA, and miRNA	Possible siRNA precipitation and aggregation or fusion of vesicles.
Detergent treatment	Selectively forms a complex with cholesterol bound to exosomal membranes to form a porous structure on the membrane surface	More loading capacity compared to electroporation	Detergents such as Saponin may show hemolysis in vivo
Freeze–thaw cycles	Repeated cycles of freezing and thawing may cause the stress-induced formation and deformation of exosomes vesicles leading to entrapment of drug during this process	Industrial acceptable technique for the preparation of the liposomes and the same can be adopted for exosomes	The drug loading capacity is usually lower than that of sonication and extrusion
Extrusion	Mechanical stress during the extrusion process may disrupt the membrane which may be resealed following the extrusion by allowing the drug to get entrapped during this process	Industrial acceptable technique for getting the liposomes with uniform distribution	However, it may damage the membrane structure of exosomes which may cause drug leakage

**Table 5:** Potential exosomal miRNAs and proteins as a biomarker for the diagnosis of breast cancer.

Sample	Method of Isolation	Biomarker	Observation	Ref.
Serum	Ultracentrifugation	miR-105	miR-105 was overexpressed in BC, which leads to the damage in tight junction and induces metastases	[76]
Serum	Precipitation	Survivin (Survivin 2B)	Survivin was high in BC patients	[77]
Breast cancer cell lines	Ultracentrifugation	HER-2	Elevated expression of HER-2 leads to resistance against Trastuzumab	[78]
Tumor tissue	Precipitation	miR-9	miR-9 promote tumor growth	[69]
Plasma	Ultracentrifugation	miR-7641	miR-7641 may promote tumor cell division and metastases	[70]
Plasma breast cancer cell lines	Precipitation	miR-1246 and miR-21	Significant high level in BC	[71]
MCF-7	Ultracentrifugation	miR-9-5p	miR-9-5p increased resistance in MCF-7 to tamoxifen	[72]
Urine	Filtration	miR-424, miR-423, miR-660, and let7-i	Significant high level in breast cancer patients when compared to normal controls	[73]
DOX-resistance PTX-resistance MCF-7 cells	Precipitation	miR-155	miR-155 increased resistance and migration capacity to normal cells	[79]
Primary epithelial BC cells from patients	Precipitation	Ex-50.T	Ex-50.T is a functional inhibitor of cellular uptake	[80]
Serum	Precipitation	Exo-XIST	Exo-XIST levels were significantly increased in TNBC patients, expression of Exo-XIST significantly reduced after resection of tumors	[81]
SK-BR-3 breast cancer cell lines	Ultracentrifugation	GAPDH, YWHAZ, and UBC	GAPDH, YWHAZ, and UBC, were overexpressed genes in BC patients	[82]
Plasma	Ultracentrifugation	miR-363-5p	The expression of miR-363-5p was downregulated in BC patients and high expression levels significantly improved in overall survival rate in BC patients	[83]
Serum	Precipitation	Small ubiquitin-like modifier 1 pseudogene 3 (SUMO1P3)	SUMO1P3 were overexpressed in TNBC patients	[84]
MDA-MB-231 and plasma	Ultracentrifugation	circPSMA1	circPSMA1 significantly overexpressed in TNBC patients and promote metastases and tumor growth both in-vivo and in-vitro	[85]
Blood	Ultracentrifugation	miR-21 and miR105	Expression of nucleotide were significantly in TNBC	[86]
Breast cancer tumors	Ultracentrifugation	miR-22 and CD63^+^ CAFs	CD63^+^ CAFs secreted exosomes contain a large amount of miR-22 which promote the tamoxifen resistance	[87]
Serum	Precipitation	IncRNA and DANCR	Expression of IncRNA and DANCr is significantly higher in BC	[74]
Bioinformatics	Gene expression omnibus	has-miR-21-5p	Expression of has-miR-21-5p is higher in BC patients	[75]

**Table 6:** The table summarizes the loading of small molecules as well as genetic materials to the exosomes and their application in breast cancer.

Cargo	Source of Exosomes	Isolation Method	Loading Method	Characteristics	Application	Ref.
**Paclitaxel**	RAW 264.7	Precipitation	Sonication and freeze–thaw cycle	Surface morphology and size was determined by AFM, NTA, DLS	To overcome MDR-cancer	[50]
	MSC	Deferential centrifugation	Co-incubation with parent cells	Particle size determined by NTA, immunoblot analysis for CD63,	Lung cancer, ovarian cancer, metastatic breast cancer	[90]
	MSC	Differential centrifugation	Extrusion	Surface morphology determined by TEM and size by NTA. Exosomal markers CD63, ALIX identified by flow cytometry	Breast cancer	[91]
**Doxorubicin**	Immature dendritic cells	Density gradient isolation	Electroporation	Surface morphology determined by TEM and size by NTA	Breast cancer	[92]
	MDA-MB-231	Precipitation	Electroporation	Surface morphology determined by TEM, TEM found spherical shape	To overcome cardiac toxicity induced by doxorubicin	[88]
	THP-1 cells	Ultracentrifugation	Incubation for overnight	Surface morphology determined by TEM and size by TNA. Exosomal markers CD81, CD63, and actin identified by Western blotting	DOX co-delivered with microRNA-155 to treat TNBC	[93]
	Electroporation	Precipitation	Electroporation	Surface morphology determined by TEM and size by Zeta, expression of MSC markers CD105 and CD90 identified by Western blotting	Improved uptake of DOX loaded exosomes by surface modification with PKH67	[94]
	Mouse macrophage cells (J774A.1)	Hybridization	Extrusion	Surface morphology determined by TEM and size by NTA	They were hybridized exosomes with liposomes to improve yield of exosomes and DOX loaded hybridized exosomes showed higher toxicity	[95]
	HEK293T cell	Precipitation	Incubation	The size was measured by NTA	Phenylboronic acid conjugated-DOX loaded exosomes showed higher cytotoxicity as compared to non-conjugated exosomes	[96]
**Olaparib**	MDA-MB-231 Cells	Precipitation	Electroporation	Surface morphology determined by TEM and size by NTA, and exosomal markers by Western blotting	Olaparib loaded exosomes were labeled with DiO, showing increased apoptosis and anti-cancer activity	[97]
**Erastin**	HFL-1	Ultracentrifugation	Sonication	Surface morphology determined by TEM and size by NTA	Targeted delivery achieved with ferritin modification, improved in apoptosis and uptake	[89]
**siS100A4**	MDA-MB-231	Ultracentrifugation	Extrusion	Particle size was measured by DLS, and exosomal biomarkers CD9, TSG101, and GRP94 identified with Western blotting	Gene silencing was achieved with target delivery and improved in vivo activity	[98]
**LNA (locked nucleic acid)-modified anti-miR-142-3p**	MSC	Precipitation	Electroporation	Particle size was measured by DLS, CD63, CD81, and Calnexin identified with Western blotting	Successfully delivered anti-miR-142-3p to silence miR-142-3p and miR-150	[99]
**miR-38-3p**	ADMSC	Filtration	Electroporation	Particle size was measured by DLS, CD63, CD81 and identified with Western blotting	Inhibition of migration, proliferation, and invasion in TNBC cells and also increase in apoptosis	[100]
**miR-424-5p**	ADMSC	Ultracentrifugation	Electroporation	Particle size was measured by NTA, CD63, CD81, and CD9 identified with Western blotting	PD-L1 downregulated by miR-424-5p, increased in the secretion of pro-inflammatory factors while decreasing the secretion of anti-inflammatory factors	[101]
**miR-33**	4T1	Precipitation	Electroporation	Morphology was determined by TEM and size by NTA	Converting M2 phase to M1 phase confirmed by specific biomarkers, increased secretion of TNF-α and IL-1β	[102]
**siMTA-1**	293T cells	Ultracentrifugation	Electroporation	--	siMTA-1 loaded exosomes improved anti-tumor activity of gemcitabine by silencing MTA1	[103]
**Exosomes**	Camel milk	Ultracentrifugation	-	Morphology was determined by TEM, kappa casein was identified by PCR	Exosomes reduced oxidative stress and immunotoxicity induced by cyclo-phosphamide	[104]

**Table 7:** A summary of clinical trials either focused on finding the suitable biomarkers for the early detection of breast cancer or as a delivery vehicle to access the therapeutic efficacy ^#^.

Trial Number	Source	Aim	Outcome	Status
**NCT04288141**	Blood samples	To identify overexpression of HER2 in tumors	HER2 overexpressed in tumors compared to HER1 and HER2	Completed
**NCT03974204**	Cerebrospinal spinal fluid	To identify leptomeningeal metastases	The cerebrospinal fluid analysis will be positive or negative	Ongoing
**NCT01344109**	Tumor	To identify biomarkers in neoadjuvant chemotherapy	-	Withdrawn
**NCT02662621**	Blood and urine samples	To identify stress proteins containing exosomes for early detection of breast cancer	Scientists are expecting that HSP70-exosomes could be a potential diagnostic marker in early detection of breast cancer and physicians in therapeutic decision making, enhancing patient care	Ongoing
**NCT04258735**	Blood samples	Genomic characterization by using exosomes in metastatic breast cancer (MBC) patients	The genomic profile will be measured in MBC patients	Ongoing
**NCT04653740**	Tumor samples before treatment and while treatment	To identify resistance to palbociclib	Changes in exosomes profile according to palbociclib will be measured	Ongoing
**NCT04530890**	Blood samples	To identify potential biomarkers in breast cancer	The composition of exosomes circulating in blood derived from tumors along with ctDNA will be measured	Ongoing
**NCT04781062**	Peripheral blood samples	Scientists are creating a non-invasive horizontal data integration (HDI) classifier from exosomes samples for early diagnosis of BC	Development of HDI will allow early detection of non-invasive breast cancer	Ongoing
**NCT02977468**	Serum	To study the effect of pembrolizumab on tumor environment in TNBC	The alteration of immune markers such as PD-L1, tumor infiltrating lymphocytes both stromal and intraepithelial will be measured in serum, tumor after treatment with pembrolizumab	Ongoing
**NCT02892734**	Blood	To study the combined effects of ipilimumab and nivolumab in inflammatory breast cancer	To assess the safety and efficacy of both the drugs by different evaluations including evaluation of ctDNA in exosomes	Ongoing
**NCT02971761**	Tumor	To study the adverse effects of pembrolizumab and enobosarm in TNBC patients	The profile of tumor-derived exosomes and associated biomarkers will be evaluated as a toxicity profile	Ongoing
**NCT04298398**	Blood	To study the effects of physiological interventions in changes in extracellular vesicles in breast cancer patients	The exosomal protein content, size, miR-21-5pa, brain RNA will be measured after mindfulness-based cognitive and emotion-focused therapy for breast cancer therapy	Ongoing

(^#^ The data was collected from the following URL; https://clinicaltrials.gov/ct2/results?cond=Breast+Cancer&term=exosomes&cntry=&state=&city=&dist=, accessed on 28 February 2022).

## Data Availability

Not applicable.

## Abbreviations

BC: Breast cancer; TNBC: Triple negative breast cancer; HER2: Human epidermal growth factor receptor 2; PR: Progesterone receptor; ER: Estrogen receptor; RES: Reticuloendeothelial system; EVs: Extracellular vesicles; DNA: Deoxyribonucleic acid; RNA: Ribonucleic acid; miRNA: microRNA; mRNA: Messenger RNA; MVBs: Multivesicular bodies; MHC: Major histocompatibility complex; ILVs: Intraluminal vesicles; ESCRT: Endosomal sorting complexes required for transport; VPS4: Vacuolar protein sorting associate protein 4; HEK239: Human embryonic kidney cell line 293; aSMase: Acid sphingomyelinase; RPE1: Retinal pigment epithelial cells 1; PBS: Phosphate buffer saline; MSC: Mesenchymal stem cells; SEC: Size-exclusion chromatography; PEG: Polyethylene glycol; PROSPR: Protein organic solvent precipitation; MTX: Methotrexate; DOX: Doxorubicin; PTX: Paclitaxel; siRNA: Small interfering RNA; BBB: Blood–brain barrier; EGFR: Epidermal growth factor receptor; TD: Transmembrane domain; BL: Before loading; AL: After loading; TEM: Transmission electron microscopy; SEM: Scanning electron microscopy; NTA: Nanoparticle tracking analysis; DLA: Dynamic light scattering; AFM: Atomic force microscopy; PCS: Photon correlation spectroscopy; EE: Entrapment efficiency; MBC: Metastatic breast cancer; SNAREs: Soluble N-ethylmaleimide-sensitive factor attachment protein receptors.
